# Small Bowel Obstruction Due to Metastatic Urachal Adenocarcinoma: A Rare Presentation

**DOI:** 10.7759/cureus.19705

**Published:** 2021-11-18

**Authors:** Pranjali Sharma, George Eigbire, Rutwik Sharma

**Affiliations:** 1 Endocrinology, Parkview Medical Center, Pueblo, USA; 2 Cardiology, Louisiana State University Health Sciences Center, New Orleans, USA; 3 Internal Medicine, Rochester Regional Health, Rochester, USA

**Keywords:** immunohistochemistry staining, chemotherapy agents, small-bowel obstruction, cancer metastasis, urachal cancer

## Abstract

Urachal adenocarcinoma is a rare but highly malignant epithelial cancer that accounts for <1% of all bladder malignancies and commonly presents with hematuria. We report a case of metastatic urachal adenocarcinoma presenting as bowel obstruction.

A 54-year-old male patient with a history of alcohol abuse presented to the emergency with acute-onset, diffuse, cramping abdominal pain, worst in the epigastrium and lasting one day. Abdominal examination revealed moderate guarding and generalized tenderness with hypoactive bowel sounds. Imaging confirmed an evolving small bowel obstruction and a urachal remnant with a superimposed mass lesion. The patient underwent an exploratory laparotomy and a high-grade small bowel obstruction due to the mass was identified. An intraoperative frozen section identified adenocarcinoma. A biopsy of the urachal mass confirmed urachal adenocarcinoma. The final diagnosis was moderately differentiated urachal adenocarcinoma. The tumor was deemed unresectable due to the involvement of multiple loops of the small bowel and the mesentery of the small and large bowels. Systemic chemotherapy with 5-fluorouracil (5-FU), leucovorin, and oxaliplatin (modified FOLFOX-6) was initiated.

Our patient did not report any prior urinary symptoms or recurrent abdominal pain, which are the common symptoms that urachal adenocarcinoma presents with. Bowel obstruction is a rare presentation of urachal adenocarcinoma since the spread of the disease to the viscera occurs much later in the course. This case report highlights a rare presentation of an even rarer malignancy.

## Introduction

Urachal carcinoma is a rare non-urothelial malignancy of the bladder that arises from the urachal ligament and constitutes less than 1% of all bladder tumors [1]. Urachal carcinoma usually presents between 47 and 56 years of age with a preponderance in men [1,2]. While it remains asymptomatic in the early stages, hematuria is the most common presenting symptom at diagnosis [2]. Bowel obstruction is an uncommon presenting symptom of urachal carcinoma. In this report, we present a case of locally metastatic urachal carcinoma with bowel obstruction as the presenting symptom.

## Case presentation

A 54-year-old male with a history of alcohol abuse presented to the emergency with acute-onset, diffuse, cramping abdominal pain, worst in the epigastrium and lasting one day. He denied fever, vomiting, loss of appetite or weight loss, diarrhea, constipation, or rectal bleeding, or any prior similar episodes. He had no prior abdominal surgeries. On examination, he was in distress and had moderate guarding and generalized tenderness with hypoactive bowel sounds. He was afebrile. Laboratory evaluation showed microcytic anemia and thrombocytopenia on complete blood count, hypokalemia on basic metabolic profile, and a normal lipase level (Table 1).

**Table 1:** Laboratory values at presentation Hb: hemoglobin; HCT: hematocrit; MCV: mean corpuscular volume; MCH: mean corpuscular hemoglobin; WBC: white blood cells; BUN: blood urea nitrogen

Labs		Normal range
Complete blood count		
Hb (gm/dl)	12.7	13.5–17.5
HCT (%)	36	41–50
MCV (fl)	100	80–100
MCH (pg)	35.3	27.5–33.2
Platelets (cells/mm^3^)	66,000	150,000–450,000
WBC (cells/mm^3^)	7,600	5,000–10,000
Basic metabolic profile		
Creatinine (mg/dl)	0.78	0.74–1.35
BUN (mg/dl)	10	8–24
Sodium (mmol/l)	140	135–145
Potassium (mmol/l)	3.4	3.6–5.2
Lipase (u/l)	30	0–160

**Figure 1:**
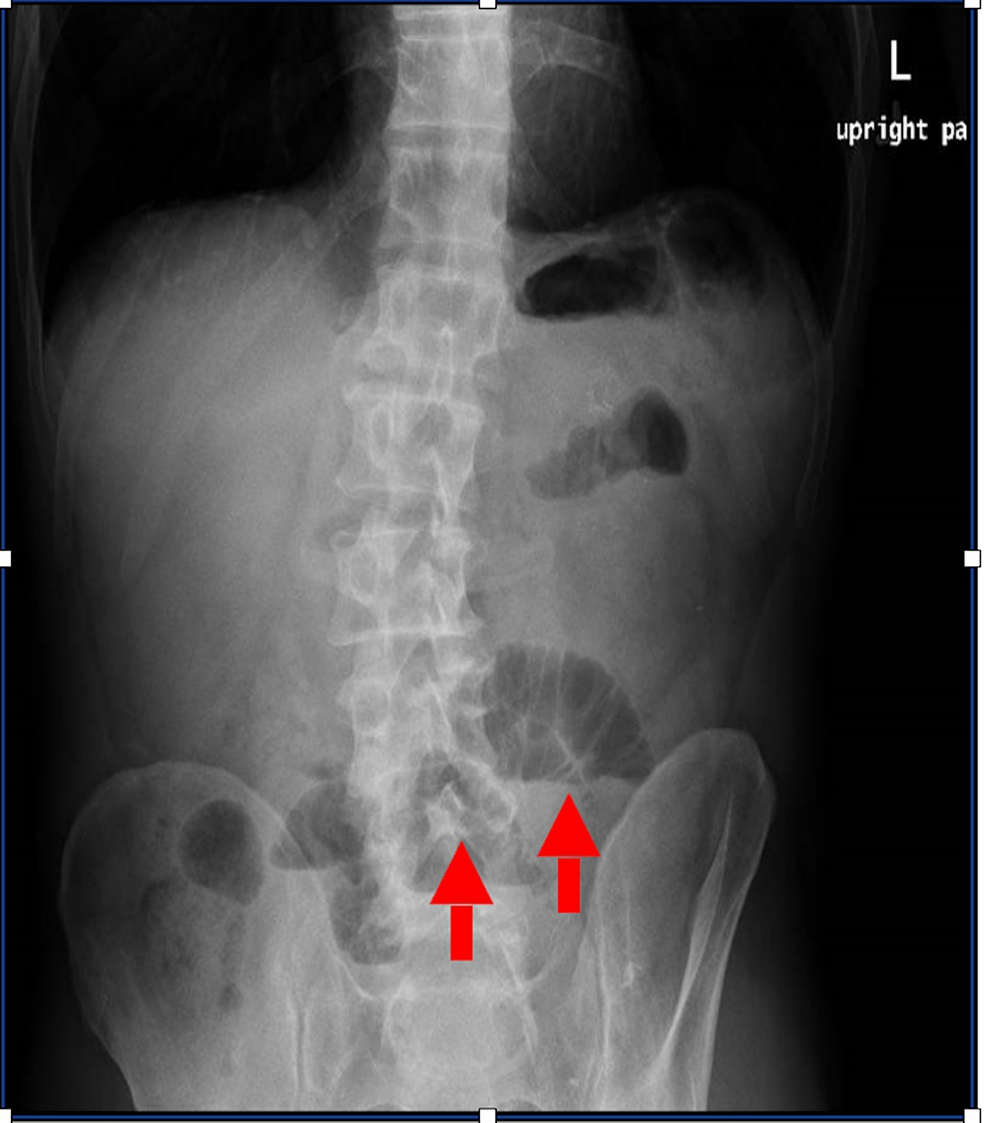
Upright abdominal X-ray Arrows indicate dilated small bowel loops that suggest evolving small bowel obstruction

**Figure 2:**
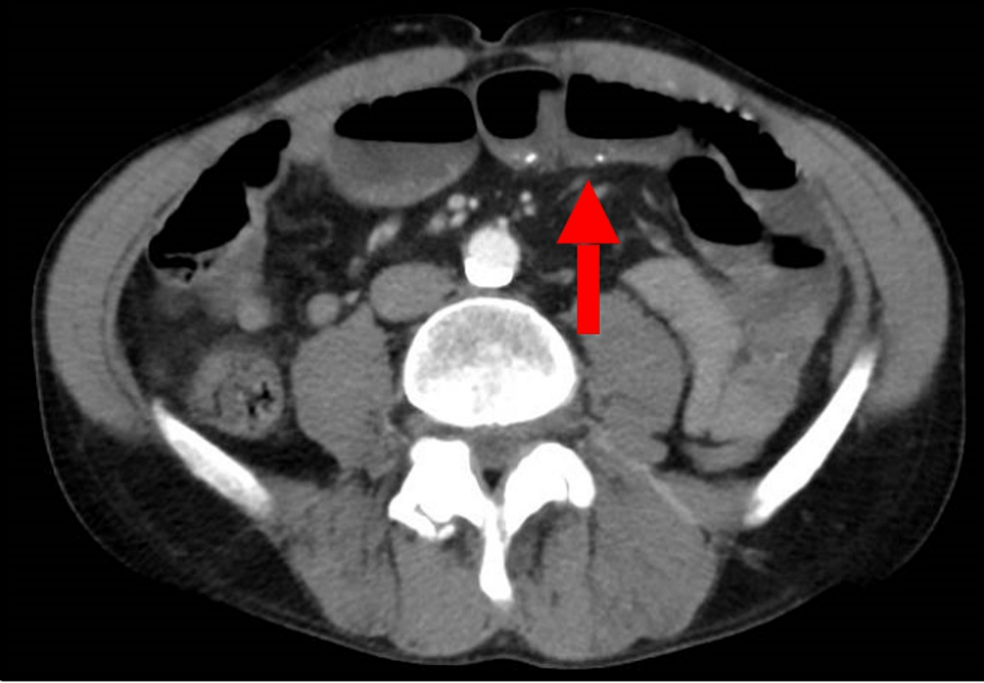
CT scan - transverse section (image 1) Arrow indicates air-fluid levels suggesting small bowel obstruction CT: computed tomography

**Figure 3:**
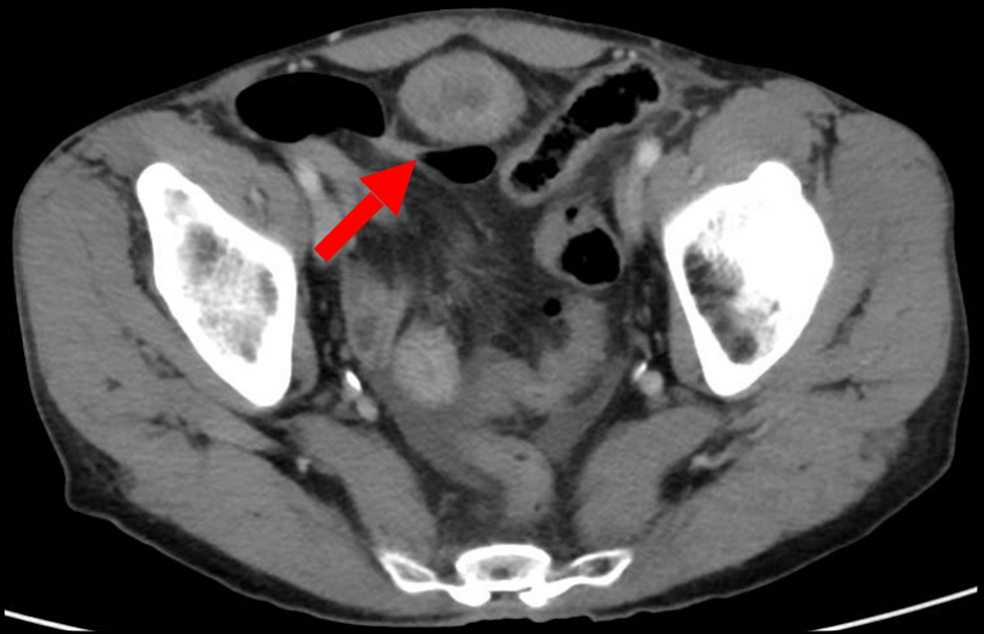
CT scan - transverse section (image 2) Arrow indicates the presence of urachal remnant CT: computed tomography

**Figure 4:**
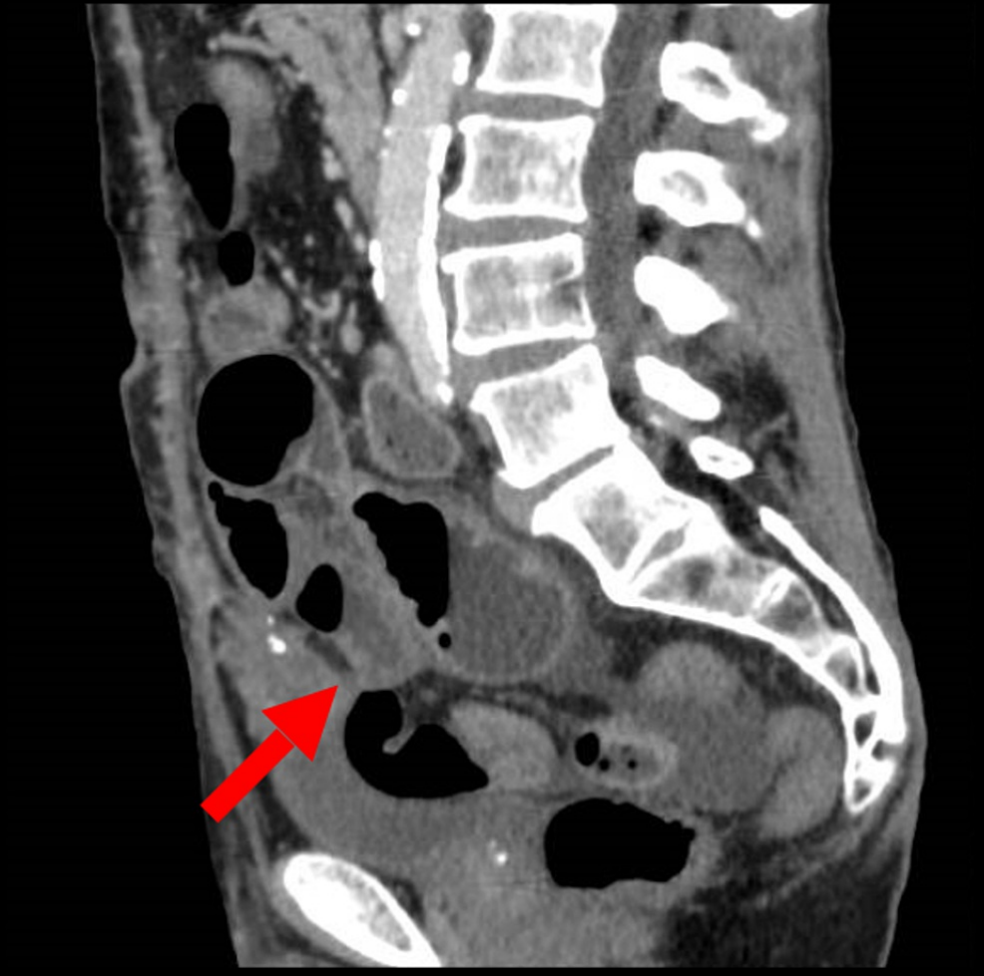
CT scan - sagittal section Arrow indicates the presence of urachal remnant CT: computed tomography

Abdominal X-ray showed an evolving small bowel obstruction (Figure 1), which was confirmed by a CT scan of the abdomen and pelvis with contrast (Figure 2). On the same CT scan series, a urachal remnant with a superimposed mass lesion was depicted (Figures 3, 4).

**Figure 5:**
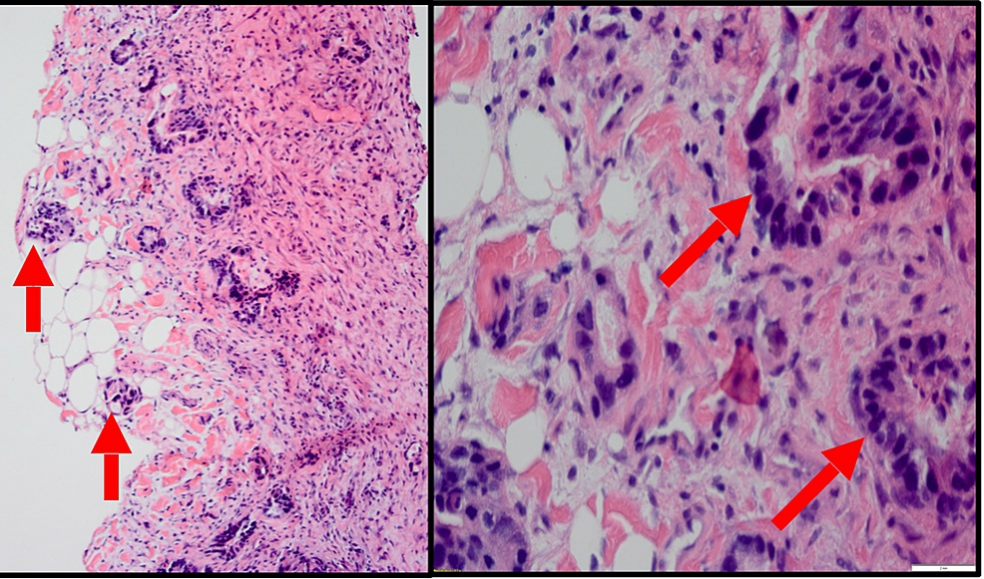
Histopathology - peritoneum [Left - low power (10x); right - high power (60x)] Arrows depict metastatic adenocarcinoma deposits in the peritoneum

A decision was made to go forward with an emergent surgery to release the small bowel obstruction. After receiving a platelet transfusion, the patient underwent an exploratory laparotomy. He was found to have a high-grade small bowel obstruction due to an extensive mass concerning for a malignancy. A frozen section was intraoperatively sent and read as adenocarcinoma (Figure 5).

**Figure 6:**
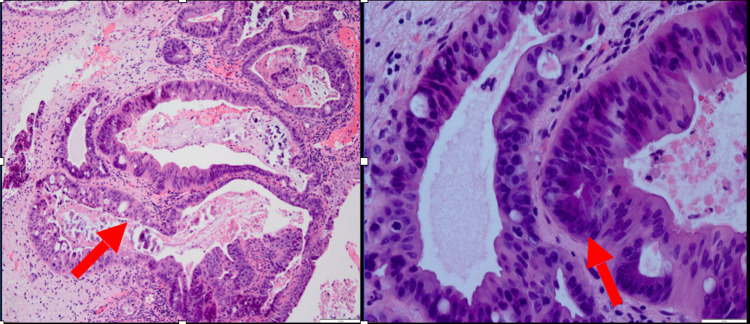
Histopathology - urachal remnant [Left - low power (10x); right - high power (100x)] Arrows indicate urachal adenocarcinoma deposits

**Figure 7:**
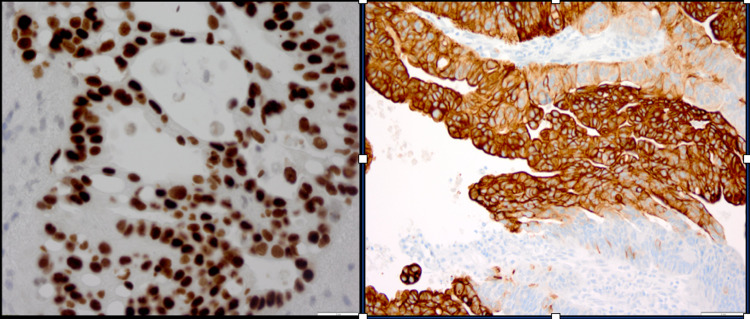
Immunohistochemistry staining - CDX2 (left), CK20 (right) The presence of brown staining in both slides suggests positive staining for CDX2 and CK20

An incisional biopsy of the urachal mass was also sent. The final pathology specimen was reported as a moderately differentiated urachal adenocarcinoma (Figure 6) with positivity for CK20, CDX2, and CK7 (focal) stains (Figure 7).

The patient was staged at Stage IIID (Sheldon staging) due to the spread to local viscera other than the bladder. The tumor was deemed unresectable due to the involvement of multiple loops of the small bowel and the mesentery of the small and large bowels. Systemic chemotherapy with 5-fluorouracil (5-FU), folinic acid, and oxaliplatin (modified FOLFOX-6) was initiated. The patient has received six cycles of chemotherapy so far. He has tolerated chemotherapy well enough and is still awaiting additional imaging to evaluate the response to therapy.

## Discussion

Urachal carcinoma is a rare non-urothelial malignancy of the bladder that arises from the urachal ligament [1]. The urachal ligament is a vestigial remnant of two embryonic structures: the cloaca (cephalic extension of the urogenital sinus that forms the bladder) and the allantois (derivative of the yolk sac) [3]. The urachal remnant may persist as a tubular or cystic structure communicating with the bladder in the midline in up to one-third of adults [1]. This can lead to congenital anomalies (patent urachus, umbilical-urachal sinus, vesicourachal diverticulum, urachal cyst) and acquired disorders (infections, benign neoplasms such as adenomas, fibromas, fibroadenomas) [3]. Our patient had no history of prior congenital anomalies. The association between the persistence of urachal remnant and the development of urachal malignancy is unclear [1]. Instead, it has been suggested that urachal carcinoma arises from the enteric rests left behind in the cloaca during embryonic development. Another theory suggests that their origin is through the metaplastic pathway [4].

The incidence of urachal carcinoma ranges between 0.35-0.7% of all bladder tumors. The most common histopathological type is adenocarcinoma, which accounts for 90% of all urachal carcinomas and 10-40% of the primary bladder adenocarcinomas [5]. It is common between the ages of 46 and 57 years with a higher preponderance in men (male-to-female ratio of 1.4-1.6:1) [2]. Our patient, who was 54 years of age, fell in the age group associated with the most common presentation of urachal carcinoma.

In the early stages, urachal carcinoma remains asymptomatic [1]. Hematuria is the most common presenting symptom at diagnosis, as seen in 58-82% of cases. Other less frequent symptoms include abdominal pain and dysuria (12-14% of cases), mucosuria (10%), pyuria, chronic urinary tract infections, abdominal or suprapubic mass, umbilical discharge, or nonspecific systemic symptoms such as nausea, fever, and weight loss [2]. Bowel obstruction is an uncommon presenting feature for urachal carcinoma. While rare in general, it is more commonly seen with a urachal cyst [6,7,8]. Our patient did not report any urinary symptoms or previous episodes of abdominal pain. His symptoms had only occurred for 24 hours prior to the presentation.

The diagnosis of urachal carcinoma is difficult due to low tumor incidence and overlapping histopathological features with adenocarcinomas of other origins [2]. Initially, strict criteria such as location in bladder dome, absence of cystitis glandularis or cystitis cystica, muscularis involvement, connection with the urachal remnant, and the presence of suprapubic mass were proposed for diagnosis [1]. However, known urachal carcinomas do not always fulfill all these criteria. Therefore, the more practical MD Anderson Cancer Center Diagnostic Criteria have now been adopted (Table 2) [1].

**Table 2:** MD Anderson Cancer Center Criteria for the diagnosis of urachal carcinoma

MD Anderson Cancer Center Diagnostic Criteria	
A	Absence of cystitis glandularis or cystitis cystica transitioning to the tumor
B	Absence of urothelial dysplasia
C	Supportive criteria:
1	Enteric type histology
2	Location in bladder dome or elsewhere in the midline of the bladder
3	Sharp demarcation between tumor and normal surface epithelium

A cystoscopy is essential for the visualization and histological diagnosis of urachal carcinoma in the midline of the bladder [1,5]. Additional imaging such as ultrasound, CT, and MRI can provide supportive evidence regarding the extent of the spread, resectability of the carcinoma, and therapeutic strategies. Staging of urachal carcinoma can be done through the older Sheldon TNM staging system or the newer Mayo staging system (Table 3) [5]. While the Sheldon staging system does not account for unusual sites, the Mayo staging system has limited application. Both systems predict cancer-related mortality equally well [5]. Our patient would have Stage III urachal adenocarcinoma due to local spread to viscera but lack of distant spread as per the Sheldon staging and Stage II urachal adenocarcinoma as per the Mayo staging.

**Table 3:** Staging of urachal carcinoma Older Sheldon staging system and newer Mayo staging system

		Sheldon staging system	Mayo staging system
I		Confined to urachal mucosa	Confined to urachus and/or bladder
II		Invasion of urachus only	Extending beyond the muscular layer of urachus/bladder
III	A	Local extension to bladder	Regional lymph node infiltration
	B	Extension to the abdominal wall	
	C	Extension to peritoneum	
	D	Extension to local viscera other than bladder	
IV	A	Metastasis to lymph nodes	Infiltration of non-regional lymph nodes or distant sites
	B	Metastasis to distant sites	

Tumor markers such as CEA, CA 125, and CA19-9 can be elevated due to peritoneal carcinomatosis, a frequent finding in metastatic disease [9]. Unfortunately, these tumor markers were not checked in our patient. Immunohistochemistry staining with CK20, as seen in our patient's histological specimen, distinguishes urachal carcinoma from non-neoplastic urachal epithelial cells [5].

In resectable cases, partial cystectomy with en-bloc resection of the urachal ligament with the umbilicus and bladder dome is done to decrease the risk of relapse [1]. Our patient's carcinoma was deemed unresectable due to the involvement of multiple loops of the bowels and the mesentery. In such unresectable cases, cisplatin and 5-FU-based regimens have shown some success. The recommended regimens for chemotherapy are listed below (Table 4) [5].

**Table 4:** Chemotherapy regimens used for urachal carcinoma 5-FU: 5-fluorouracil

Chemotherapy regimens	
First-line	Gemcitabine + cisplatin (GP)
	5-FU + cisplatin (FP)
	Paclitaxel + cisplatin (TP)
	Methotrexate + vinblastine + doxorubicin + cisplatin (MVAC)
	Methotrexate + vinblastine + cisplatin (CMV)
	Etoposide + ifosfamide + cisplatin (VIP)
Second-line	Paclitaxel + carboplatin (TC)
	Etoposide + cisplatin (EP)
	Bleomycin + vincristine + mitomycin + cisplatin (BOMP)
	Etoposide + ifosfamide (VI)
	Paclitaxel
Other regimens	5-FU
	Oxaliplatin + 5-FU + leucovorin (modified FOLFOX-6)
	5-FU + leucovorin + irinotecan
	Ifosfamide + 5-FU
	Etoposide + cisplatin
	Irinotecan

Our patient has received six cycles of the modified FOLFOX-6 regimen and has tolerated it so far. However, tumors often recur and the majority of the patients die within two years of the diagnosis [10].

## Conclusions

Urachal carcinoma is a rare but highly malignant tumor that is difficult to diagnose clinically. While urinary complaints such as hematuria and dysuria or abdominal mass are the more common presenting symptoms, in rare cases, the clinical picture could be more severe. This was the clinical presentation of our patient. He had experienced no symptoms until he presented with bowel obstruction due to metastatic urachal carcinoma. Our case highlights a rare presentation of an even rarer malignancy.
